# Association of maternal HDL2-c concentration in the first trimester and the risk of large for gestational age birth

**DOI:** 10.1186/s12944-022-01688-w

**Published:** 2022-08-15

**Authors:** Dongxu Huang, Haiyan Zhu, Yandi Zhu, Qinyu Dang, Qian Yang, Yadi Zhang, Xiaxia Cai, Xiaoyan Zhao, Ning Liang, Hongliang Wang, Huanling Yu

**Affiliations:** 1 Department of Nutrition and Food Hygiene, School of Public Health, Capital Medical University, No.10, Xitoutiao, Youanmenwai, Fengtai District, Beijing, 100069 People’s Republic of China; 2 Obstetrical Department, Fuxing Hospital, Capital Medical University, Beijing, 100045 People’s Republic of China

**Keywords:** Birthweight, HDL, HDL subfractions, LGA, Maternal lipids

## Abstract

**Background:**

Maternal lipid levels during pregnancy are critical for fetal development. Recent studies revealed that high-density lipoprotein cholesterol (HDL-c) levels during pregnancy were negatively correlated with birthweight. High-density lipoprotein 2 cholesterol (HDL2-c) is one of the major subclasses of HDL-c, and its relationship with birthweight is unclear. Association of HDL2-c concentration in the first trimester and risk of large for gestational age (LGA) was explored.

**Methods:**

This study recruited pregnant women who registered in Fuxing Hospital from October 2018 to January 2020, had regular obstetric examinations during pregnancy, and delivered between June 2019 and September 2020. Finally, 549 participants were recruited for the study. Maternal demographic characteristics and venous blood were collected at the 6th-14th gestational week, and serum total cholesterol (TC), triglyceride (TG), HDL-c, HDL2-c, high-density lipoprotein 3 cholesterol (HDL3-c), and low-density lipoprotein cholesterol (LDL-c) concentrations were detected. Neonatal characteristics were collected at delivery. A logistic regression model was used to explore the relationship between the first trimester HDL2-c concentration and LGA incidence. A nomogram was developed, and the performance was evaluated with a concordance index.

**Results:**

Seventy-five mothers delivered LGA infants, and the LGA incidence was 13.66%. LGA mothers had significantly lower serum HDL-c and HDL2-c concentrations than appropriate for gestational age (AGA) mothers. A logistic regression model showed that HDL2-c concentration was negatively correlated with LGA risk (odds ratio (OR) = 0.237, 95% confidence intervals (CI): 0.099–0.567, *P* = 0.001) when adjusted for age, prepregnancy body mass index (BMI), and parity. A nomogram was generated using all these risk factors. The area under the curve (AUC) was 0.663 (95% CI: 0.593–0.732).

**Conclusions:**

Maternal HDL2-c concentration in the first trimester was negatively correlated with the risk of LGA.

**Supplementary Information:**

The online version contains supplementary material available at 10.1186/s12944-022-01688-w.

## Background

High-density lipoprotein cholesterol (HDL-c) refers to cholesterol and cholesterol esters carried by HDL particles, and recently researchers found that maternal HDL-c concentrations were negatively correlated with birthweight. Misra et al. [1] found that birthweight was negatively correlated with HDL-c concentrations after the 10th week of gestation. A study by our team revealed a negative relationship between birthweight and HDL-c levels at 24th and 36th weeks of gestation [2]. A meta-analysis showed that HDL-c concentrations were reversely correlated with birthweight throughout pregnancy, especially in the third trimester [3]. In summary, maternal HDL-c levels throughout gestation were negatively associated with birthweight.

HDL particles are heterogeneous and consist of multiple subcomponents of different sizes and densities. Based on the difference in density, HDL can be divided into HDL2 and HDL3 by ultracentrifugation. HDL2 has a larger size, smaller density, and weaker antioxidant capacity than HDL3. Whether high-density lipoprotein 2 cholesterol (HDL2-c) or high-density lipoprotein 3 cholesterol (HDL3-c) plays a significant role in fetal growth and birthweight is unclear. However, some evidence suggests that HDL2-c concentration may be a critical factor. A longitudinal study showed that the lengths and head circumferences of newborns correlated negatively with the proportion of HDL2a subclasses in mothers’ plasma before delivery [4]. Another study found that compared with mothers of full-term infants, mothers of preterm infants had higher large HDL concentrations among black women [5]. Similar associations have not been reported for maternal HDL3-c levels.

Large for gestational age (LGA) refers to those newborns whose birthweight are higher than the 90th percentile of the mean birthweight or 2 standard deviations above the mean birthweight of neonates with the same gestational age. Recently, the incidence of LGA has been increasing in China, reaching 8.2%-17.7% in different regions [6]. LGA causes adverse pregnancy outcomes, such as obstructed labor [7] and neonatal asphyxia [8], as well as metabolic diseases in childhood and adulthood [9]. Recent studies found that higher HDL-c concentration was associated with a lower risk of LGA/macrosomia. Research on Chinese individuals found that increased HDL-c concentrations in the mid-pregnancy were correlated with a lower risk for macrosomia [10]. A correlation was noted between decreased HDL-c concentrations and an increased risk of LGA/macrosomia based on meta-analysis [3]. However, whether HDL2-c concentration correlates with LGA incidence has not yet been determined. In addition, early pregnancy is a critical period for fetal development, and it is of great importance to pay attention to maternal lipid levels in the early pregnancy and explore its relationship with fetal development to avoid adverse pregnancy outcomes. The study explored the correlation between HDL2-c concentration in the first trimester and birthweight using the incidence of LGA as the primary outcome measure.

## Methods

### Study design

This study recruited pregnant women who registered at Fuxing Hospital from October 2018 to January 2020, had regular obstetric examinations during pregnancy, and delivered between June 2019 and September 2020 as the research population. The criteria for inclusion and exclusion are as follows. The following inclusion criteria were employed: 1) 20–40 years of age; 2) singleton pregnancy; 3) natural fertilization; and 4) first blood collection was performed before the 14th gestational week. The following exclusion criteria were employed: 1) women with infectious disease or other severe disease; 2) fetal malformation or birth defects; and 3) Apgar score < 7 at the 5th min.

Finally, 549 participants were recruited for the study.

### Data collection

A questionnaire survey was conducted to obtain maternal demographic characteristics at their first hospital visit. Data collected included age, height, prepregnancy weight, gravidity, parity, disease history, education background and occupation. Neonatal data at delivery, including newborn sex, birthweight, birth length, gestational weeks, gestational weight gain (GWG), mode of delivery and perinatal outcome, were collected in this study.

### Measurement of maternal blood lipids

Fasting blood samples of pregnant women were collected for measurement of total cholesterol (TC), triglyceride (TG), low-density lipoprotein cholesterol (LDL-c), and HDL-c serum concentrations at the 6th-14th weeks. The HDL3-c concentration was measured using a single precipitation method [11]. In brief, 0.06 ml of precipitation reagent, which consisted of heparin (8.25 mg/ml), MnCl_2_ (98.7 mg/ml), and dextran sulfate (12 mg/ml), was added to 0.3 ml of serum. The mixture was settled at room temperature for 30 min, and centrifuged at 10,000 rpm at 4 °C for 10 min. An aliquot of the supernatant was taken for HDL3-c measurement. To correct for reagent dilution, the HDL3-c value was multiplied by 1.2. Value of HDL2-c concentration was calculated by subtracting HDL3-c from HDL-c.

### Statistical analysis

Data were analyzed by SPSS 26.0 and R software. The independent sample t test and chi-square test were used to analyze the differences between the appropriate for gestational age (AGA) and LGA groups. Kendall’s tau_b correlation was used to analyze the associations between LGA incidence and maternal concentrations of HDL-c, HDL2-c, and HDL3-c as well as the ratio of HDL2-c/HDL3-c. The logistic regression model was adjusted based on maternal age, prepregnancy body mass index (BMI), gestational weight gain and parity. A *P* value < 0.05 was defined as significantly different. A nomogram for LGA risk was created based on the logistic regression model. The nomogram performance was evaluated by a concordance index.

## Results

### Maternal and neonatal characteristics

In total, 75 mothers delivered LGA infants among all 549 pregnant women, and the LGA incidence was 13.66%. The average age, prepregnancy BMI and GWG of the pregnant women were 31.4 ± 3.7 years old, 21.84 ± 2.95 kg/m^2^ and 13.25 ± 4.97 kg in the LGA and AGA groups, and no significant difference was detected. The average birth weight, head circumference and birth length of LGA group were significantly higher than those of AGA group, as expected. In terms of parity, neonatal sex, as well as mode of delivery, no significant difference was detected. All the results were shown in Table 1.

**Table 1:** Maternal and neonatal characteristics

	Total (*n* = 549)	AGA (*n* = 458)	LGA (*n* = 75)	*P* value^a^
Maternal characteristics				
Age (years)	31.4 ± 3.7	31.5 ± 3.7	31.3 ± 3.2	0.687
Prepregnancy BMI (kg/m^2^)	21.84 ± 2.95	21.77 ± 2.96	22.27 ± 2.78	0.169
GWG (kg)	13.25 ± 4.97	13.09 ± 4.93	14.25 ± 5.20	0.066
Parity				0.861
1	375(68.3)	310(67.7)	50(66.7)	
> 1	174(31.7)	148(32.3)	25(33.3)	
Neonatal characteristics				
Gender				0.279
Male	295(53.7)	244(53.3)	45(60.0)	
Female	254(46.3)	214(46.7)	30(40.0)	
Birth length (cm)	49.57 ± 1.82	49.41 ± 1.72	51.03 ± 1.57	0.000
Birth weight (g)	3348.67 ± 411.95	3277.79 ± 327.26	3936.67 ± 307.06	0.000
Birth head circumference (cm)	34.66 ± 1.20	34.55 ± 1.11	35.69 ± 1.05	0.000
Delivery mode				0.337
Vaginal delivery	381(70.6)	318(70.8)	49(65.3)	
Cesarean section	159(29.4)	131(29.2)	26(34.7)	

*AGA* Appropriate for gestational age, *LGA* Large for gestational age, *GWG* Gestational weight gain, *BMI* Body mass index

^a^ Statistically significant difference between AGA and LGA groups

### Association of HDL2-c concentration in the first trimester and LGA incidence

**Fig. 1:**
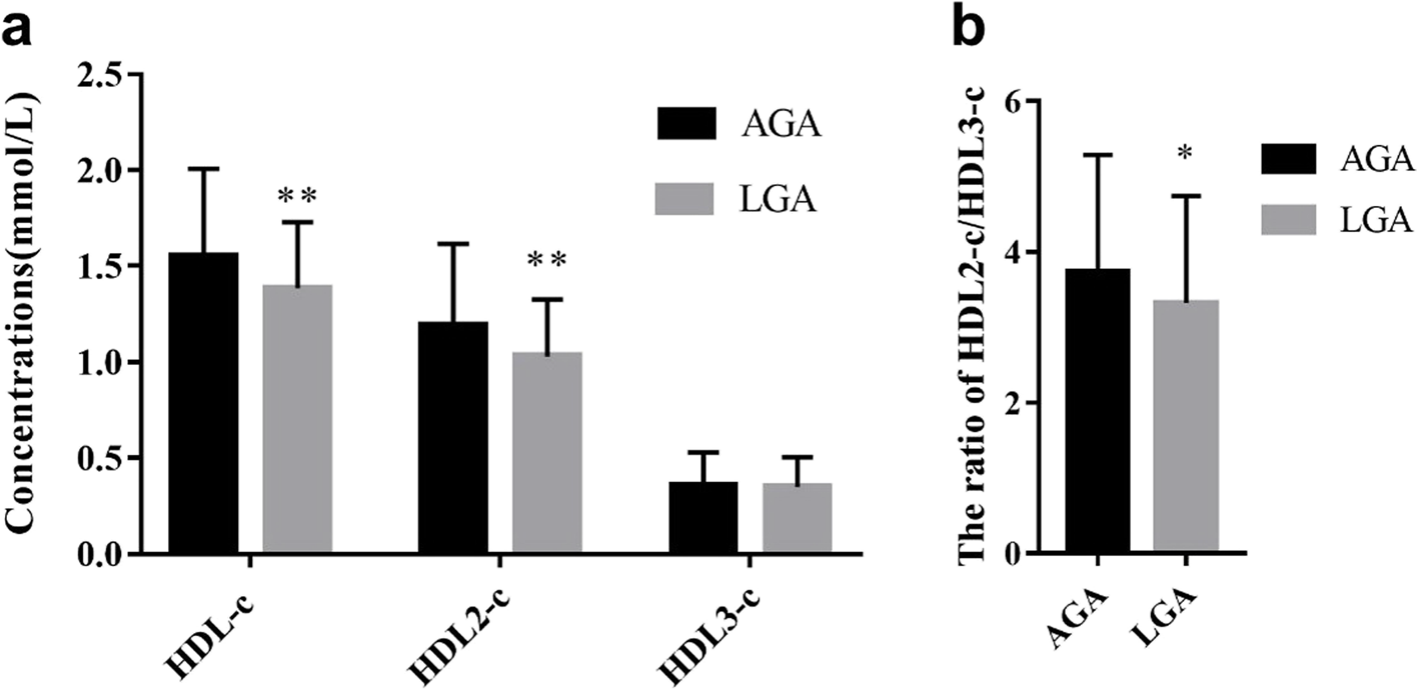
Maternal serum HDL-c, HDL2-c, and HDL3-c concentrations and HDL2-c/HDL3-c ratios in the first trimester in the AGA and LGA groups. **a** Maternal serum HDL-c, HDL2-c, and HDL3-c concentrations in the first trimester in the AGA and LGA groups. **b** The ratio of HDL2-c/HDL3-c in the AGA and LGA groups. AGA, appropriate for gestational age. LGA, large for gestational age. *P < 0.05, **P < 0.01

Compared to AGA mothers, LGA mothers had significantly lower serum HDL-c (1.384 ± 0.345 mmol/L vs. 1.553 ± 0.454 mmol/L) and HDL2-c concentrations (1.031 ± 0.296 mmol/L vs. 1.193 ± 0.423 mmol/L) (Fig. 1a) as well as a lower ratio of HDL2-c/HDL3-c (3.984 ± 1.710 vs. 4.484 ± 1.863) in the first trimester (Fig. 1b).

**Fig. 2:**
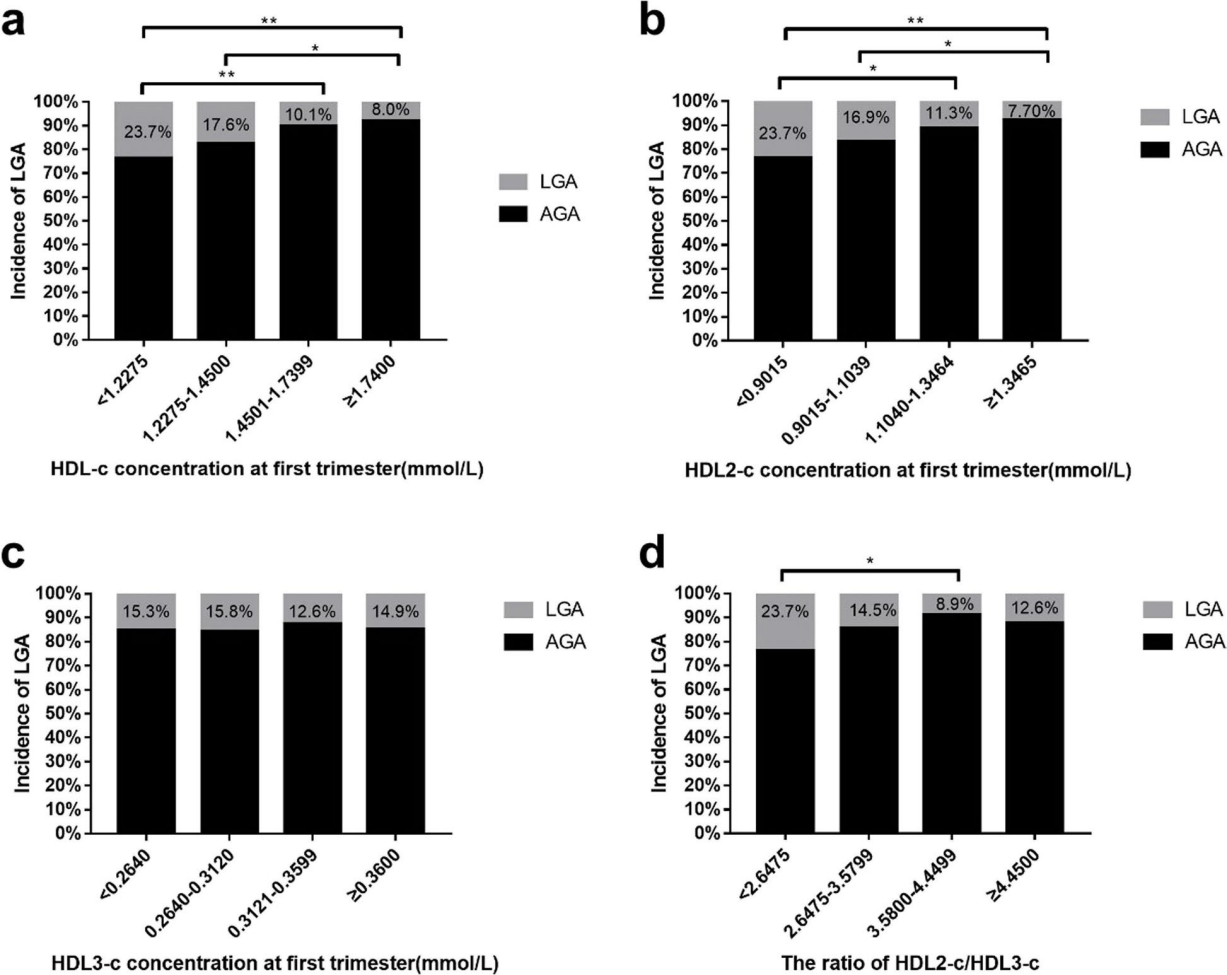
The incidence of LGA based on different maternal HDL-c, HDL2-c, and HDL3-c concentrations and HDL2-c/HDL3-c ratios in the first trimester. **a** The incidence of LGA based on different maternal HDL-c concentrations in the first trimester. **b** The incidence of LGA based on different maternal HDL2-c concentrations in the first trimester. **c** The incidence of LGA based on different maternal HDL3-c concentrations in the first trimester. **d** The incidence of LGA based on different maternal ratios of HDL2-c/HDL3-c in the first trimester. LGA, large for gestational age. AGA, appropriate for gestational age. *P value < 0.05, **P value < 0.01

HDL-c, HDL2-c and HDL3-c concentrations and the ratio of HDL2-c/HDL3-c were grouped into quartiles. Compared to the group with the lowest level of HDL-c (HDL-c < 1.2275 mmol/L), the LGA incidence in the two groups with the highest HDL-c levels (1.4501 ≤ HDL-c ≤ 1.7399 mmol/L and HDL-c ≥ 1.7400 mmol/L) were significantly lower (*P* < 0.01, *P* < 0.01) (Fig. 2a). Compared to the group with the lowest level of HDL2-c (HDL2-c < 0.9015 mmol/L), the LGA incidence in the two groups with the highest HDL2-c levels (1.1040 ≤ HDL2-c ≤ 1.3464 mmol/L and HDL-c ≥ 1.3465 mmol/L) was significantly lower (*P* < 0.05, *P* < 0.01) (Fig. 2b). Compared to the group with the lowest level of the ratio of HDL2-c/HDL3-c (ratio of HDL2-c/HDL3-c < 2.6475), the LGA incidence in the group with the ratio of HDL2-c/HDL3-c (3.5800 ≤ HDL2-c/HDL3-c ≤ 4.4499) was significantly lower (*P* < 0.05) (Fig. 2d).

Kendall’s tau_b correlations were used to explore the association between the incidence of LGA and HDL-c, HDL2-c, and HDL3-c concentrations as well as the ratio of HDL2-c/HDL3-c. Concentrations of HDL-c, HDL2-c and HDL3-c and the ratio of HDL2-c/HDL3-c were grouped into quartiles. Table 2 shows that the HDL-c and HDL2-c concentrations and the ratio of HDL2-c/HDL3-c were negatively associated with LGA incidence (*P* < 0.01, *P* < 0.01, *P* < 0.05), yet no correlation was found between HDL3-c concentration and the LGA incidence.

**Table 2:** Kendall's tau_b correlations between the incidence of LGA and HDL-c, HDL2-c, and HDL3-c concentrations as well as the ratio of HDL2-c/HDL3-c

Kendall's tau_b	HDL-c	HDL2-c	HDL3-c	HDL2-c/HDL3-c
Correlation coefficient	-0.124^**^	-0.129^**^	-0.011	-0.089^*^
*P* value	0.001	0.001	0.773	0.020

*LGA* Large for gestational age. ^*^P < 0.05, ^**^P < 0.01

**Fig. 3:**
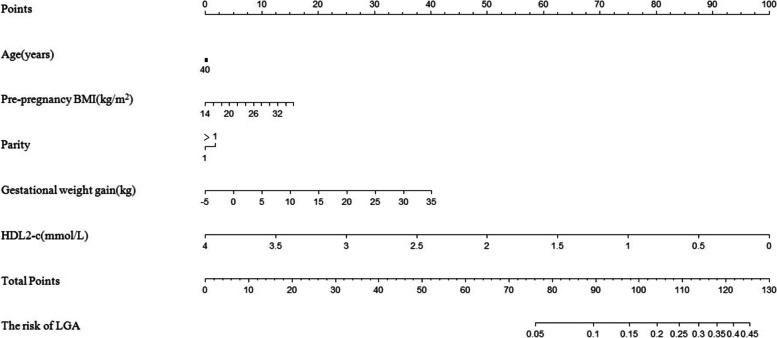
Nomogram for the risk of LGA. To estimate the probability of LGA, the values of a pregnant woman value were marked at each axis. A straight line was drawn perpendicular to the point axis, and the points for all variables were summed. Next, the sum was noted on the total point axis, and a straight line was drawn perpendicular to the probability axis. LGA, large for gestational age. GWG, gestational weight gain. BMI, body mass index

**Fig. 4:**
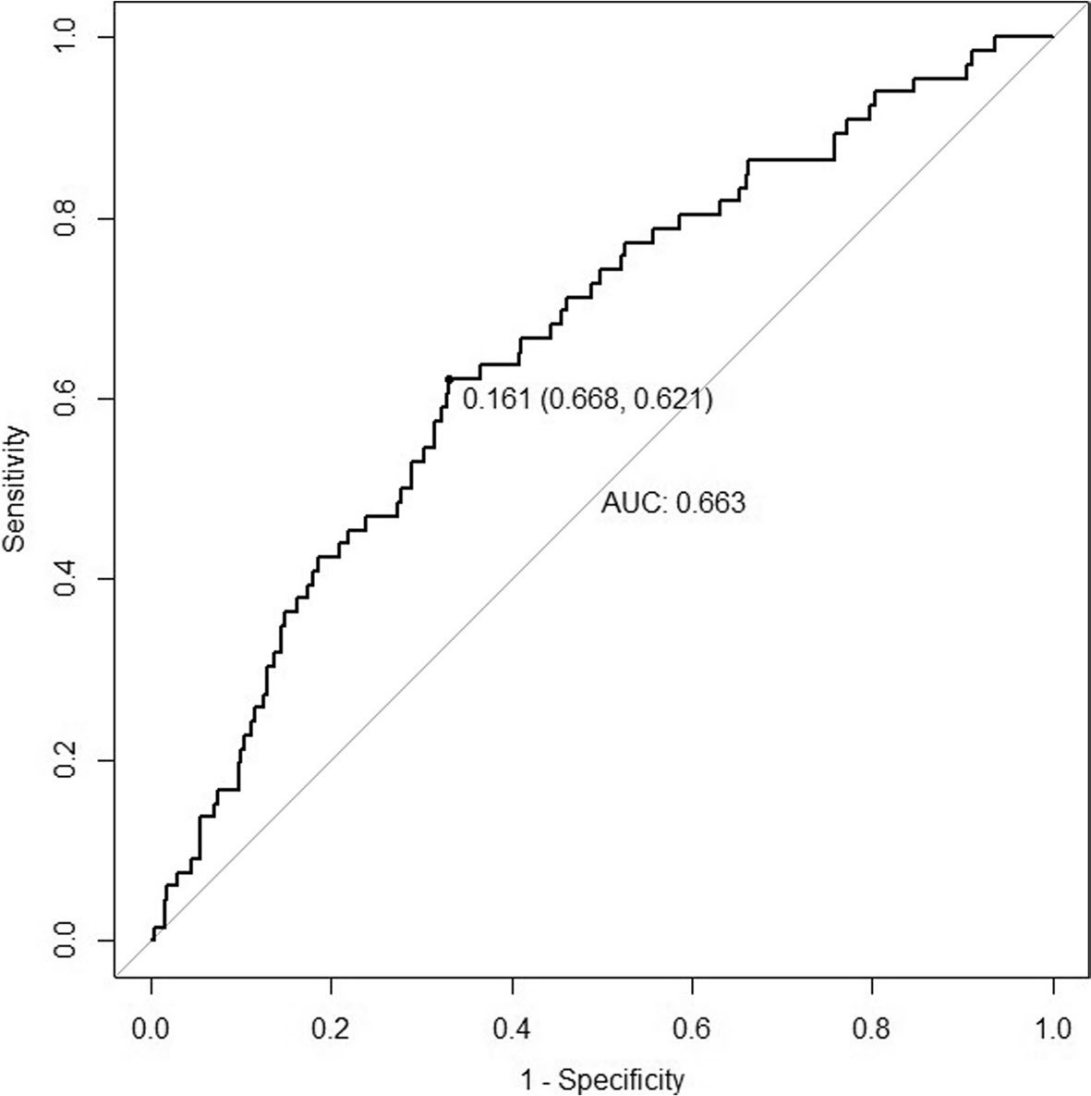
Receiver operating characteristic curve for the prediction model. The area under the curve (AUC) was 0.663 (95% CI 0.593–0.732)

A Logistic regression was performed to explore the association between maternal HDL2-c concentration in the first trimester and the risk of LGA. The model was adjusted by maternal age, pre-BMI, GWG, and parity. HDL2-c concentration (OR = 0.237, *P* = 0.001) was a protective factor for LGA. A 1 mmol/L increase in HDL2-c concentration was associated with a 23.7% decrease in the incidence of LGA (95% CI 0.099–0.567). GWG (OR = 1.059, *P* = 0.034) was positively associated with the risk of LGA (Table 3). Then, a nomogram was created using all these factors (Fig. 3). The area under the curve (AUC) was 0.663 (95% CI 0.593–0.732) (Fig. 4).

**Table 3:** The association between maternal HDL2-c concentration at first trimester and risk of LGA

Variables	OR	95% CI for OR	*P* value
Age (year)	0.996	0.918–1.080	0.919
GWG (kg)	1.059	1.004–1.116	0.034
Prepregnancy BMI (kg/m^2^)			
18.5–23.9	Reference		
< 18.5	0.544	0.157–1.883	0.336
≥ 24	1.097	0.559–2.151	0.788
Parity			
1	Reference		
> 1	1.134	0.606–2.122	0.695
HDL2-c (mmol/L)	0.237	0.099–0.567	0.001

The model was adjusted for maternal age, GWG, prepregnancy BMI and parity. *GWG* Gestational weight gain, *BMI* Body mass index

## Discussion

HDL is the predominant lipoprotein in follicular fluid (FF). FF provides cholesterol for steroid production [12], and regulates intrafollicular cholesterol homeostasis [13]. HDL improves oocyte quality and early embryonic development, which may be related to the antioxidant defense capacity of ApoAI and PON1 [14, 15]. However, recent studies have shown that maternal HDL-c levels throughout the gestation were inversely correlated with newborns’ birth weight. Misra et al. [1] found that birthweight was negatively correlated with HDL-c concentrations after the 10th week of gestation. Each 1 mg/dl increase in maternal HDL-c concentration was correlated with a 6.4 g reduction in birthweight in mothers with normal weight and a 13 g reduction in birthweight in those who were overweight or obese. According to a previous study by our team, birthweight was negatively correlated with maternal HDL-c concentrations in the middle and late pregnancies. Small for gestational age (SGA) mothers had higher HDL-c concentrations at 16–20 gestational weeks compared to AGA mothers [16], whereas LGA mothers had lower HDL-c concentrations in the third gestation [17]. Research on the Chinese pregnant women showed that low HDL-c levels were correlated with higher risk of macrosomia as well as lower incidence of SGA [10]. In this study, LGA mothers had significantly lower serum HDL-c concentrations than AGA mothers, and maternal HDL-c concentration was negatively associated with the risk of LGA. These findings were consistent with previous studies.

Based on the difference in density, HDL can be divided into HDL2 and HDL3 by ultracentrifugation. Density of HDL2 was 1.063–1.125 g/ml and density of HDL3 was of 1.125–1.210 g/ml. HDL2 has a larger size and weaker antioxidant capacity than HDL3 [18]. Although whether HDL2 or HDL3 plays a critical role in fetal growth and birthweight is unclear, some evidence suggests that HDL2-c concentration may be a critical factor. During pregnancy, the HDL2b proportion increased greatly, representing the most predominant subfraction in late pregnancy, which may be associated with estrogen [19]. A study found that the mothers of preterm infants had higher large HDL concentrations than those of full-term infants in black women [5]. Another study also revealed that mothers of macrosomia had significantly lower HDL2-c concentrations than mothers with AGA infants in the first and third trimesters, regardless of prepregnancy BMI [20]. A longitudinal study showed that the lengths and head circumferences of newborns correlated negatively with the proportion of the HDL2a subclass in mothers’ plasma before delivery [4].

In the present study, HDL2-c concentrations of LGA mothers were significantly lower than those of AGA mothers, but HDL3-c concentrations didn’t differ between two groups. In addition, a negative correlation between HDL2-c concentration and the risk of LGA was found in this study. The logistic regression model showed that when adjusted by maternal age, prepregnancy BMI, and parity, HDL2-c concentration was negatively correlated with the risk of LGA (OR = 0.237, 95% CI: 0.099–0.567, *p* = 0.001). Each 1 mmol/L increase in HDL2-c concentration decreased the risk of LGA by 23.7%. The study also created a nomogram for the risk of LGA using the factors included in the logistic regression model: age, prepregnancy BMI, parity, GWG and HDL2-c concentration in the first trimester. A concordance index was used to evaluate nomogram performance and the AUC was 0.693, indicating a certain discriminative ability. The nomogram suggested that high GWG and low HDL2-c concentrations were the major risk factors for LGA.

The decrease in total antioxidant capacity of HDL particles may be the mechanism involved in the association between HDL2-c levels and the proportion of HDL2-c and fetal development. HDL antioxidant function is mainly realized by ApoA-I, PON1, PAF-AH and other components. Studies have found that ApoA-I is more enriched in small and dense HDL3-c compared to HDL2a and HDL2b [21], and PON1 is mainly present in the HDL3 subclass [22]. In addition, PAF-AH enzymatic activity is also preferentially localized in HDL3. The antioxidant activity of HDL subclasses decreases with density : HDL3c > HDL3b > HDL3a > HDL2b > HDL2a [23]. Decreased HDL3-c concentrations were strongly correlated with an higher risk of cardiovascular diseases (for example, coronary heart disease) and death, whereas HDL2 lacked such associations [24]. The proportion of HDL2b increases significantly during pregnancy and becomes the predominant HDL subcomponent in the third trimester [19]. During pregnancy, the levels of serum lipids increase, and oxidative stress in the body increases. HDL particles with normal physiological functions can reduce the level of oxidative stress through antioxidant effects. With high HDL2-c levels, the total antioxidant capacity of HDL particles decreases, resulting in the inability to effectively suppress oxidative stress levels. A systematic review showed that the serum antioxidant capacity of pregnant women who delivered fetal growth restriction neonates was attenuated and that oxidative stress was enhanced [25]. When HDL2-c levels and its proportion increase, the total antioxidant capacity of HDL particles decreases, which is not conducive to fetal growth and development. From another perspective, it also reduces the risk of LGA.

In recent years, the incidence of LGA in infants has been increasing in China, reaching 8.2%-17.7% in different regions. The incidence of LGA in our study was 13.66%. Women who delivered LGA infants are more likely to have pregnancy complications, including cephalopelvic disproportion and postpartum hemorrhage. Regarding birth outcomes, LGA infants were more likely to get shoulder dystocia, neonatal injury, birth asphyxia and neonatal death. LGA was confirmed to be associated with maternal hyperglycemia, hypertriglyceridemia, obesity, excessive GWG and advanced age. Although the results showed a reverse association between maternal cholesterol levels and LGA incidence, it is important to control cholesterol levels during pregnancy given that abnormal elevation showed an adverse effect on birthweight, which may result in fetal growth restriction, low birth weight and SGA.

### Comparisons with other studies and what does the current work add to the existing knowledge

Previous studies have typically focused on TC and HDL-c levels in mid-pregnancy or late pregnancy and explored their relationship with birthweight. The present study focused on maternal HDL2-c concentrations in the first trimester and found a negative association with the risk of LGA.

### Study strength and limitations

The present study not only found a negative association between maternal HDL-c concentration and LGA incidence, but also revealed that maternal HDL2-c concentration in the first trimester was negatively associated with the risk of LGA. However, this study had some limitations. First, when exploring the association between HDL2-c concentration and birthweight, SGA infants were excluded. In addition, information on pregnant women lifestyle wasn’t collected. For instance, pregnancy diet and physical activity, and these may be confounders. It is necessary to explore the relationship between maternal HDL2-c concentration and SGA incidence to elucidate its effect on birthweight, and maternal lifestyle should be taken into account in these studies.

## Conclusion

In conclusion, high maternal HDL-c and HDL2-c levels in the first trimester were negatively correlated with the risk of LGA. For pregnant women, it is important to detect and monitor maternal HDL2-c concentrations in the early pregnancy to evaluate embryonic and fetal development and avoid adverse birth outcomes.

## Supplementary Information

**Additional file 1.**

**Additional file 2.**

**Additional file 3.**

## Data Availability

The datasets analyzed during the current study are not publicly available because they are also part of an ongoing study but are available from the corresponding author on reasonable request.

## Abbreviations

AGA: Appropriate for gestational age
AUC: Area under the curve
BMI: Body mass index
CI: Confidence intervals
FF: Follicular fluid
GWG: Gestational weight gain
HDL-c: High-density lipoprotein cholesterol
HDL2-c: High-density lipoprotein 2 cholesterol
HDL3-c: High-density lipoprotein 3 cholesterol
LDL-c: Low-density lipoprotein cholesterol
LGA: Large for gestational age
OR: Odds ratio
SGA: Small for gestational age
TC: Total cholesterol
TG: Triglyceride
