# Supplementary material for: Association of maternal HDL2-c concentration in the first trimester and the risk of large for gestational age birth
Source: Lipids Health Dis. 2022 Aug 15;21:71. doi: 10.1186/s12944-022-01688-w (PMC9380360; doi:10.1186/s12944-022-01688-w)
Supplement: Supplementary file 1 — Additional file 1. [file 12944_2022_1688_MOESM1_ESM.pdf]

Editing Certificate

This document certifies that the manuscript

Association of maternal HDL2-c concentration at first trimester and the risk of large for gestational age birth

prepared by the authors

Dongxu Huang, Haiyan Zhu, Yandi Zhu, Qinyu Dang, Qian Yang, Yadi Zhang, Xiaxia Cai, Xiaoyan Zhao, Ning Liang, Hongliang Wang, Huanling Yu

was edited for proper English language, grammar, punctuation, spelling, and overall style by one or more of the highly qualified native English speaking editors at AJE.

This certificate was issued on **July 30, 2022** and may be verified on the AJE website using the verification code **E6D8-C761-E402-E255-C6CD**.

Neither the research content nor the authors' intentions were altered in any way during the editing process. Documents receiving this certification should be English-ready for publication; however, the author has the ability to accept or reject our suggestions and changes. To verify the final AJE edited version, please visit our verification page at aje.com/certificate. If you have any questions or concerns about this edited document, please contact AJE at support@aje.com.

AJE provides a range of editing, translation, and manuscript services for researchers and publishers around the world. For more information about our company, services, and partner discounts, please visit aje.com.
