# Supplementary material for: Association of maternal HDL2-c concentration in the first trimester and the risk of large for gestational age birth
Source: Lipids Health Dis. 2022 Aug 15;21:71. doi: 10.1186/s12944-022-01688-w (PMC9380360; doi:10.1186/s12944-022-01688-w)
Supplement: Supplementary file 3 — Additional file 3. [file 12944_2022_1688_MOESM3_ESM.pdf]

20220803105359752159393072443392

WORD COUNT 4505
TIME SUBMITTED 02-AUG-2022 10:54PM
PAPER ID 89124024

Title: Association of maternal HDL2-c concentration in the first trimester and the risk of large for gestational age birth

Dongxu Huang [a], Haiyan Zhu [b], Yandi Zhu [a], Qinyu Dang [a], Qian Yang [a], Yadi Zhang [a], Xiaxia Cai [a], Xiaoyan Zhao [a], Ning Liang [a], Hongliang Wang [a], Huanling Yu [a*]

[a] Department of Nutrition and Food Hygiene, School of Public Health, Capital Medical University, Beijing 100069, P.R. China

[b] Obstetrical Department, Fuxing Hospital, Capital Medical University, Beijing 100045, P.R. China

*** Corresponding author**

Huanling Yu, Professor

Department of Nutrition and Food Hygiene, School of Public Health, Capital Medical University, No.10, Xitoutiao, Youanmenwai, Fengtai District, Beijing 100069, P.R. China

Tel./Fax: +86-010-83911652, E-mail address: yuhlzjl@ccmu.edu.cn

Author's E-mails

Dongxu Huang: 17801061801@163.com

Haiyan Zhu: Zhuhaiyan929@126.com

Yandi Zhu: zhuyandi209@126.com

Qinyu Dang: Dang_qinyu@163.com

Qian Yang: syyq0903@163.com

Yadi Zhang: xgzzyd@126.com

Xiaxia Cai: caixx1988@ccmu.edu.cn

Xiaoyan Zhao: zxy_escapist@sina.com

Ning Liang: lningice@126.com

Hongliang Wang: andrea0417@126.com

Huanling Yu: yuhlzjl@ccmu.edu.cn

Number of pages (including Figure legends): 22

Number of figures: 4

Number of tables: 3

ABSTRACT

Background: Maternal lipid levels during pregnancy are critical for fetal development. Recent studies revealed that high-density lipoprotein cholesterol (HDL-c) levels during pregnancy were negatively correlated with birthweight. High-density lipoprotein 2 cholesterol (HDL2-c) is one of the major subclasses of HDL-c, and its relationship with birthweight is unclear. Association of HDL2-c concentration in the first trimester and risk of large for gestational age (LGA) was explored.

Methods: This study recruited pregnant women who registered in Fuxing Hospital from October 2018

to January 2020, had regular obstetric examinations during pregnancy, and delivered between June 2019 and September 2020. Finally, 549 participants were recruited for the study. Maternal demographic characteristics and venous blood were collected at the 6th-14th gestational week, and serum total cholesterol (TC), triglyceride (TG), HDL-c, HDL2-c, high-density lipoprotein 3 cholesterol (HDL3-c), and low-density lipoprotein cholesterol (LDL-c) concentrations were detected. Neonatal characteristics were collected at delivery. A logistic regression model was used to explore the relationship between the first trimester HDL2-c concentration and LGA incidence. A nomogram was developed, and the performance was evaluated with a concordance index.

Results: Seventy-five mothers delivered LGA infants, and the LGA incidence was 13.66%. LGA mothers had significantly lower serum HDL-c and HDL2-c concentrations than appropriate for gestational age (AGA) mothers. A logistic regression model showed that HDL2-c concentration was negatively correlated with LGA risk (odds ratio (OR)=0.237, 95% confidence intervals (CI): 0.099-0.567, *P*=0.001) when adjusted for age, prepregnancy body mass index (BMI), and parity. A nomogram was generated using all these risk factors. The area under the curve (AUC) was 0.663 (95% CI: 0.593-0.732).

Conclusions: Maternal HDL2-c concentration in the first trimester was negatively correlated with the risk of LGA.

Key words: Birthweight, HDL, HDL subfractions, LGA, Maternal lipids

BACKGROUND

High-density lipoprotein cholesterol (HDL-c) refers to cholesterol and cholesterol esters carried by HDL particles, and recently researchers found that maternal HDL-c concentrations were negatively correlated with birthweight. Misra et al.[1] found that birthweight was negatively correlated with HDL-c concentrations after the 10th week of gestation. A study by our team revealed a negative relationship between birthweight and HDL-c levels at 24th and 36th weeks of gestation[2]. A meta-analysis showed that HDL-c concentrations were reversely correlated with birthweight throughout pregnancy, especially in the third trimester[3]. In summary, maternal HDL-c levels throughout gestation were negatively associated with birthweight.

HDL particles are heterogeneous and consist of multiple subcomponents of different sizes and densities. Based on the difference in density, HDL can be divided into HDL2 and HDL3 by ultracentrifugation. HDL2 has a larger size, smaller density, and weaker antioxidant capacity than HDL3. Whether high-density lipoprotein 2 cholesterol (HDL2-c) or high-density lipoprotein 3 cholesterol (HDL3-c) plays a significant role in fetal growth and birthweight is unclear. However, some evidence suggests that HDL2-c concentration may be a critical factor. A longitudinal study showed that the lengths and head circumferences of newborns correlated negatively with the proportion of HDL2a subclasses in mothers' plasma before delivery[4]. Another study found that compared with mothers of full-term infants, mothers of preterm infants had higher large HDL concentrations among black women[5]. Similar associations have not been reported for maternal HDL3-c levels.

Large for gestational age (LGA) refers to those newborns whose birthweight are higher than the

90th percentile of the mean birthweight or 2 standard deviations above the mean birthweight of neonates with the same gestational age. Recently, the incidence of LGA has been increasing in China, reaching 8.2%-17.7% in different regions[6]. LGA causes adverse pregnancy outcomes, such as obstructed labor[7] and neonatal asphyxia[8], as well as metabolic diseases in childhood and adulthood[9]. Recent studies found that higher HDL-c concentration was associated with a lower risk of LGA/macrosomia. Research on Chinese individuals found that increased HDL-c concentrations in the mid-pregnancy were correlated with a lower risk for macrosomia[10]. A correlation was noted between decreased HDL-c concentrations and an increased risk of LGA/macrosomia based on meta-analysis[3]. However, whether HDL2-c concentration correlates with LGA incidence has not yet been determined. In addition, early pregnancy is a critical period for fetal development, and it is of great importance to pay attention to maternal lipid levels in the early pregnancy and explore its relationship with fetal development to avoid adverse pregnancy outcomes. The study explored the correlation between HDL2-c concentration in the first trimester and birthweight using the incidence of LGA as the primary outcome measure.

METHODS

Study design

This study recruited pregnant women who registered at Fuxing Hospital from October 2018 to January 2020, had regular obstetric examinations during pregnancy, and delivered between June 2019 and September 2020 as the research population. The criteria for inclusion and exclusion are as follows. The following inclusion criteria were employed: 1) 20-40 years of age; 2) singleton pregnancy; 3)

natural fertilization; and 4) first blood collection was performed before the 14th gestational week. The following exclusion criteria were employed: 1) women with infectious disease or other severe disease; 2) fetal malformation or birth defects; and 3) Apgar score<7 at the 5th min.

Finally, 549 participants were recruited for the study.

Data collection

A questionnaire survey was conducted to obtain maternal demographic characteristics at their first hospital visit. Data collected included age, height, prepregnancy weight, gravidity, parity, disease history, education background and occupation. Neonatal data at delivery, including newborn sex, birthweight, birth length, gestational weeks, gestational weight gain (GWG), mode of delivery and perinatal outcome, were collected in this study.

Measurement of maternal blood lipids

Fasting blood samples of pregnant women were collected for measurement of total cholesterol (TC), triglyceride (TG), low-density lipoprotein cholesterol (LDL-c), and HDL-c serum concentrations at the 6th-14th weeks. The HDL3-c concentration was measured using a single precipitation method[11]. In brief, 0.06 ml of precipitation reagent, which consisted of heparin (8.25 mg/ml), $MnCl_2$ (98.7 mg/ml), and dextran sulfate (12 mg/ml), was added to 0.3 ml of serum. The mixture was settled at room temperature for 30 min, and centrifuged at 10,000 rpm at 4 °C for 10 min. An aliquot of the supernatant was taken for HDL3-c measurement. To correct for reagent dilution, the HDL3-c value was multiplied by 1.2. Value of HDL2-c concentration was calculated by subtracting HDL3-c from HDL-c.

Statistical analysis

Data were analyzed by SPSS 26.0 and R software. The independent sample t test and chi-square test were used to analyze the differences between the appropriate for gestational age (AGA) and LGA groups. Kendall's tau_b correlation was used to analyze the associations between LGA incidence and maternal concentrations of HDL-c, HDL2-c, and HDL3-c as well as the ratio of HDL2-c/HDL3-c. The logistic regression model was adjusted based on maternal age, prepregnancy body mass index (BMI), gestational weight gain and parity. A *P* value <0.05 was defined as significantly different. A nomogram for LGA risk was created based on the logistic regression model. The nomogram performance was evaluated by a concordance index.

RESULTS

Maternal and neonatal characteristics

In total, 75 mothers delivered LGA infants among all 549 pregnant women, and the LGA incidence was 13.66%. The average age, prepregnancy BMI and GWG of the pregnant women were 31.4 ± 3.7 years old, 21.84 ± 2.95 kg/m^2 and 13.25 ± 4.97 kg in the LGA and AGA groups, and no significant difference was detected. The average birth weight, head circumference and birth length of LGA group were significantly higher than those of AGA group, as expected. In terms of parity, neonatal sex, as well as mode of delivery, no significant difference was detected. All the results were shown in **Table 1**.

Table 1 Maternal and neonatal characteristics

	Total (n=549)	AGA (n=458)	LGA (n=75)	*P* value[a]
Maternal characteristics				

Age (years)	31.4±3.7	31.5±3.7	31.3±3.2	0.687
Prepregnancy BMI (kg/m^2)	21.84±2.95	21.77±2.96	22.27±2.78	0.169
GWG (kg)	13.25±4.97	13.09±4.93	14.25±5.20	0.066
Parity				0.861
1	375(68.3)	310(67.7)	50(66.7)	
>1	174(31.7)	148(32.3)	25(33.3)	
Neonatal characteristics				
Gender				0.279
Male	295(53.7)	244(53.3)	45(60.0)	
Female	254(46.3)	214(46.7)	30(40.0)	
Birth length (cm)	49.57±1.82	49.41±1.72	51.03±1.57	0.000
Birth weight (g)	3348.67±411.95	3277.79±327.26	3936.67±307.06	0.000
Birth head circumference (cm)	34.66±1.20	34.55±1.11	35.69±1.05	0.000
Delivery mode				0.337
Vaginal delivery	381(70.6)	318(70.8)	49(65.3)	
Cesarean section	159(29.4)	131(29.2)	26(34.7)	

AGA, appropriate for gestational age. LGA, large for gestational age. GWG, gestational weight gain. BMI, body mass index.

[a] Statistically significant difference between AGA and LGA groups.

Association of HDL2-c concentration in the first trimester and LGA incidence

Compared to AGA mothers, LGA mothers had significantly lower serum HDL-c (1.384 ± 0.345

mmol/L vs. 1.553 ± 0.454 mmol/L) and HDL2-c concentrations (1.031 ± 0.296 mmol/L vs. 1.193 ± 0.423 mmol/L) (**Figure 1 a**) as well as a lower ratio of HDL2-c/HDL3-c (3.984 ± 1.710 vs. 4.484 ± 1.863) in the first trimester (**Figure 1 b**).

HDL-c, HDL2-c and HDL3-c concentrations and the ratio of HDL2-c/HDL3-c were grouped into quartiles. Compared to the group with the lowest level of HDL-c (HDL-c < 1.2275 mmol/L), the LGA incidence in the two groups with the highest HDL-c levels (1.4501 ≤ HDL-c ≤ 1.7399 mmol/L and HDL-c ≥ 1.7400 mmol/L) were significantly lower ($P < 0.01$, $P < 0.01$) (**Figure 2 a**). Compared to the group with the lowest level of HDL2-c (HDL2-c < 0.9015 mmol/L), the LGA incidence in the two groups with the highest HDL2-c levels (1.1040 ≤ HDL2-c ≤ 1.3464 mmol/L and HDL-c ≥ 1.3465 mmol/L) was significantly lower ($P < 0.05$, $P < 0.01$) (**Figure 2 b**). Compared to the group with the lowest level of the ratio of HDL2-c/HDL3-c (ratio of HDL2-c/HDL3-c < 2.6475), the LGA incidence in the group with the ratio of HDL2-c/HDL3-c (3.5800 ≤ HDL2-c/HDL3-c ≤ 4.4499) was significantly lower ($P < 0.05$) (**Figure 2 d**).

Kendall's tau_b correlations were used to explore the association between the incidence of LGA and HDL-c, HDL2-c, and HDL3-c concentrations as well as the ratio of HDL2-c/HDL3-c. Concentrations of HDL-c, HDL2-c and HDL3-c and the ratio of HDL2-c/HDL3-c were grouped into quartiles. **Table 2** shows that the HDL-c and HDL2-c concentrations and the ratio of HDL2-c/HDL3-c were negatively associated with LGA incidence ($P < 0.01$, $P < 0.01$, $P < 0.05$), yet no correlation was found between HDL3-c concentration and the LGA incidence.

Table 2 Kendall's tau_b correlations between the incidence of LGA and HDL-c, HDL2-c, and HDL3-c concentrations as well as the ratio of HDL2-c/HDL3-c

Kendall's tau_b	HDL-c	HDL2-c	HDL3-c	HDL2-c/HDL3-c
Correlation coefficient	-0.124**	-0.129**	-0.011	-0.089*
P value	0.001	0.001	0.773	0.020

LGA, large for gestational age. *$P < 0.05$, **$P < 0.01$

A Logistic regression was performed to explore the association between maternal HDL2-c concentration in the first trimester and the risk of LGA. The model was adjusted by maternal age, pre-BMI, GWG, and parity. HDL2-c concentration (OR=0.237, *P*=0.001) was a protective factor for LGA. A 1 mmol/L increase in HDL2-c concentration was associated with a 23.7% decrease in the incidence of LGA (95% CI 0.099-0.567). GWG (OR=1.059, *P*=0.034) was positively associated with the risk of LGA (**Table 3**). Then, a nomogram was created using all these factors (**Figure 3**). The area under the curve (AUC) was 0.663 (95% CI 0.593-0.732) (**Figure 4**).

Table 3 The association between maternal HDL2-c concentration at first trimester and risk of LGA

Variables	OR	95% CI for OR	*P* value
Age (year)	0.996	0.918-1.080	0.919
GWG (kg)	1.059	1.004-1.116	0.034
Prepregnancy BMI (kg/m^2)			
18.5-23.9	Reference		
<18.5	0.544	0.157-1.883	0.336
≥24	1.097	0.559-2.151	0.788

Parity			
1	Reference		
>1	1.134	0.606-2.122	0.695
HDL2-c (mmol/L)	0.237	0.099-0.567	0.001

The model was adjusted for maternal age, GWG, prepregnancy BMI and parity. GWG, gestational weight gain. BMI, body mass index.

DISCUSSION

HDL is the predominant lipoprotein in follicular fluid (FF). FF provides cholesterol for steroid production[12], and regulates intrafollicular cholesterol homeostasis[13]. HDL improves oocyte quality and early embryonic development, which may be related to the antioxidant defense capacity of ApoAI and PON1[14, 15]. However, recent studies have shown that maternal HDL-c levels throughout the gestation were inversely correlated with newborns' birth weight. Misra et al.[1] found that birthweight was negatively correlated with HDL-c concentrations after the 10th week of gestation. Each 1 mg/dl increase in maternal HDL-c concentration was correlated with a 6.4 g reduction in birthweight in mothers with normal weight and a 13 g reduction in birthweight in those who were overweight or obese. According to a previous study by our team, birthweight was negatively correlated with maternal HDL-c concentrations in the middle and late pregnancies. Small for gestational age (SGA) mothers had higher HDL-c concentrations at 16-20 gestational weeks compared to AGA mothers[16], whereas LGA mothers had lower HDL-c concentrations in the third gestation[17]. Research on the Chinese pregnant women showed that low HDL-c levels were correlated with higher risk of macrosomia as well as lower

incidence of SGA[10]. In this study, LGA mothers had significantly lower serum HDL-c concentrations than AGA mothers, and maternal HDL-c concentration was negatively associated with the risk of LGA. These findings were consistent with previous studies.

Based on the difference in density, HDL can be divided into HDL2 and HDL3 by ultracentrifugation. Density of HDL2 was 1.063-1.125 g/ml and density of HDL3 was of 1.125-1.210 g/ml. HDL2 has a larger size and weaker antioxidant capacity than HDL3[18]. Although whether HDL2 or HDL3 plays a critical role in fetal growth and birthweight is unclear, some evidence suggests that HDL2-c concentration may be a critical factor. During pregnancy, the HDL2b proportion increased greatly, representing the most predominant subfraction in late pregnancy, which may be associated with estrogen[19]. A study found that the mothers of preterm infants had higher large HDL concentrations than those of full-term infants in black women[5]. Another study also revealed that mothers of macrosomia had significantly lower HDL2-c concentrations than mothers with AGA infants in the first and third trimesters, regardless of prepregnancy BMI[20]. A longitudinal study showed that the lengths and head circumferences of newborns correlated negatively with the proportion of the HDL2a subclass in mothers' plasma before delivery[4].

In the present study, HDL2-c concentrations of LGA mothers were significantly lower than those of AGA mothers, but HDL3-c concentrations didn't differ between two groups. In addition, a negative correlation between HDL2-c concentration and the risk of LGA was found in this study. The logistic regression model showed that when adjusted by maternal age, prepregnancy BMI, and parity, HDL2-c concentration was negatively correlated with the risk of LGA (OR=0.237, 95% CI: 0.099-0.567,

p=0.001). Each 1 mmol/L increase in HDL2-c concentration decreased the risk of LGA by 23.7%. The study also created a nomogram for the risk of LGA using the factors included in the logistic regression model: age, prepregnancy BMI, parity, GWG and HDL2-c concentration in the first trimester. A concordance index was used to evaluate nomogram performance and the AUC was 0.693, indicating a certain discriminative ability. The nomogram suggested that high GWG and low HDL2-c concentrations were the major risk factors for LGA.

The decrease in total antioxidant capacity of HDL particles may be the mechanism involved in the association between HDL2-c levels and the proportion of HDL2-c and fetal development. HDL antioxidant function is mainly realized by ApoA-I, PON1, PAF-AH and other components. Studies have found that ApoA-I is more enriched in small and dense HDL3-c compared to HDL2a and HDL2b[21], and PON1 is mainly present in the HDL3 subclass[22]. In addition, PAF-AH enzymatic activity is also preferentially localized in HDL3. The antioxidant activity of HDL subclasses decreases with density : HDL3c > HDL3b > HDL3a > HDL2b > HDL2a[23]. Decreased HDL3-c concentrations were strongly correlated with an higher risk of cardiovascular diseases (for example, coronary heart disease) and death, whereas HDL2 lacked such associations[24]. The proportion of HDL2b increases significantly during pregnancy and becomes the predominant HDL subcomponent in the third trimester[19]. During pregnancy, the levels of serum lipids increase, and oxidative stress in the body increases. HDL particles with normal physiological functions can reduce the level of oxidative stress through antioxidant effects. With high HDL2-c levels, the total antioxidant capacity of HDL particles decreases, resulting in the inability to effectively suppress oxidative stress levels. A systematic review showed that the serum

antioxidant capacity of pregnant women who delivered fetal growth restriction neonates was attenuated and that oxidative stress was enhanced[25]. When HDL2-c levels and its proportion increase, the total antioxidant capacity of HDL particles decreases, which is not conducive to fetal growth and development. From another perspective, it also reduces the risk of LGA.

In recent years, the incidence of LGA in infants has been increasing in China, reaching 8.2%-17.7% in different regions. The incidence of LGA in our study was 13.66%. Women who delivered LGA infants are more likely to have pregnancy complications, including cephalopelvic disproportion and postpartum hemorrhage. Regarding birth outcomes, LGA infants were more likely to get shoulder dystocia, neonatal injury, birth asphyxia and neonatal death. LGA was confirmed to be associated with maternal hyperglycemia, hypertriglyceridemia, obesity, excessive GWG and advanced age. Although the results showed a reverse association between maternal cholesterol levels and LGA incidence, it is important to control cholesterol levels during pregnancy given that abnormal elevation showed an adverse effect on birthweight, which may result in fetal growth restriction, low birth weight and SGA.

Comparisons with other studies and what does the current work add to the existing knowledge

Previous studies have typically focused on TC and HDL-c levels in mid-pregnancy or late pregnancy and explored their relationship with birthweight. The present study focused on maternal HDL2-c concentrations in the first trimester and found a negative association with the risk of LGA.

Study strength and limitations

The present study not only found a negative association between maternal HDL-c concentration and LGA incidence, but also revealed that maternal HDL2-c concentration in the first trimester was

negatively associated with the risk of LGA. However, this study had some limitations. First, when exploring the association between HDL2-c concentration and birthweight, SGA infants were excluded. In addition, information on pregnant women lifestyle wasn't collected. For instance, pregnancy diet and physical activity, and these may be confounders. It is necessary to explore the relationship between maternal HDL2-c concentration and SGA incidence to elucidate its effect on birthweight, and maternal lifestyle should be taken into account in these studies.

CONCLUSION

In conclusion, high maternal HDL-c and HDL2-c levels in the first trimester were negatively correlated with the risk of LGA. For pregnant women, it is important to detect and monitor maternal HDL2-c concentrations in the early pregnancy to evaluate embryonic and fetal development and avoid adverse birth outcomes.

List of Abbreviations

AGA, Appropriate for gestational age

AUC, Area under the curve

BMI, Body mass index

CI, Confidence intervals

FF, Follicular fluid

GWG, Gestational weight gain

HDL-c, High-density lipoprotein cholesterol

HDL2-c, High-density lipoprotein 2 cholesterol

HDL3-c, High-density lipoprotein 3 cholesterol

LDL-c, Low-density lipoprotein cholesterol

LGA, Large for gestational age

OR, Odds ratio

SGA, Small for gestational age

TC, Total cholesterol

TG, Triglyceride

Declarations

Ethics approval and consent to participate

The study was approved in accordance with the requirements of the Ethics Committee of Capital Medical University (2018SY04). Informed consent for the scientific use of biological materials was obtained from all patients before enrollment.

Consent for publication

Not applicable.

Availability of data and materials

The datasets analyzed during the current study are not publicly available because they are also part of an ongoing study but are available from the corresponding author on reasonable request.

Competing interests

The authors declare they have no competing interests with respect to this research study and paper.

Funding

This work was supported by the National Natural Science Foundation of China [No. 8187120380].

Authors' contributions

HLY and DXH designed the study. QYD, YDZ and DXH analyzed the data. DXH drafted the manuscript. HYZ, QY, XXC, NL, HLW, XYZ, and YDZ helped in collecting blood samples and data. All authors read and approved the final manuscript.

Acknowledgments

We thank the participants in the study for their support and the staff of the laboratory and Obstetrical Department at Fuxing Hospital for their help.

References

1. Misra VK, Trudeau S, Perni U. Maternal serum lipids during pregnancy and infant birth weight: the influence of prepregnancy BMI. Obesity. 2011;19(7):1476-1481.
2. Wang H, Dang Q, Zhu H, et al. Associations between maternal serum HDL-c concentrations during pregnancy and neonatal birth weight: a population-based cohort study. Lipids Health Dis. 2020;19(1):93.
3. Wang J, Moore D, Subramanian A, et al. Gestational dyslipidaemia and adverse birthweight outcomes: a systematic review and meta-analysis. Obes Rev. 2018;19(9):1256-1268.

4. Zeljkovic A, Vekic J, Spasic S, et al. Changes in LDL and HDL subclasses in normal pregnancy and associations with birth weight, birth length and head circumference. Matern Child Health J. 2013;17(3):556-565.

5. Catov JM, Mackey RH, Scifres CM, Bertolet M, Simhan HN. Lipoprotein Heterogeneity Early in Pregnancy and Preterm Birth. Am J Perinatol. 2017;34(13):1326-1332.

6. Zhu L, et al. Chinese neonatal birth weight curve for different gestational age. Zhonghua Er Ke Za Zhi. 2015;53(2):97-103.

7. Spellacy WN, Miller S, Winegar A, Peterson PQ. Macrosomia--maternal characteristics and infant complications. Obstet Gynecol. 1985;66(2):158-161.

8. Boulet SL, Salihu HM, Alexander GR. Mode of delivery and birth outcomes of macrosomic infants. J Obstet Gynaecol. 2004;24(6):622-629.

9. Eriksson J, Forsén T, Tuomilehto J, Osmond C, Barker D. Size at birth, childhood growth and obesity in adult life. Int J Obes Relat Metab Disord. 2001;25(5):735-740.

10. Jin WY, Lin SL, Hou RL, et al. Associations between maternal lipid profile and pregnancy complications and perinatal outcomes: a population-based study from China. BMC Pregnancy Childbirth. 2016;16:60.

11. Hirano T, Nohtomi K, Koba S, Muroi A, Ito Y. A simple and precise method for measuring HDL-cholesterol subfractions by a single precipitation followed by homogenous HDL-cholesterol assay. J Lipid Res. 2008;49(5):1130-1136.

12. Jaspard B, Collet X, Barbaras R, et al. Biochemical characterization of pre-beta 1 high-density

lipoprotein from human ovarian follicular fluid: evidence for the presence of a lipid core. Biochemistry. 1996;35(5):1352-1357.

13. Fujimoto VY, Kane JP, Ishida BY, Bloom MS, Browne RW. High-density lipoprotein metabolism and the human embryo. Hum Reprod Update. 2010;16(1):20-38.

14. Browne RW, Shelly WB, Bloom MS, et al. Distributions of high-density lipoprotein particle components in human follicular fluid and sera and their associations with embryo morphology parameters during IVF. Hum Reprod. 2008;23(8):1884-1894.

15. Rincón J, Madeira EM, Campos FT, et al. Exogenous paraoxonase-1 during oocyte maturation improves bovine embryo development in vitro. Reprod Domest Anim. 2016;51(5):827-830.

16. Kramer MS, Kahn SR, Dahhou M, et al. Maternal lipids and small for gestational age birth at term. J Pediatr. 2013;163(4):983-988.

17. Hou RL, Zhou HH, Chen XY, Wang XM, Shao J, Zhao ZY. Effect of maternal lipid profile, C-peptide, insulin, and HBA1c levels during late pregnancy on large-for-gestational age newborns. World J Pediatr. 2014;10(2):175-181.

18. Brites F, Martin M, Guillas I, Kontush A. Antioxidative activity of high-density lipoprotein (HDL): Mechanistic insights into potential clinical benefit. BBA Clin. 2017;8:66-77.

19. Alvarez JJ, Montelongo A, Iglesias A, Lasunción MA, Herrera E. Longitudinal study on lipoprotein profile, high density lipoprotein subclass, and postheparin lipases during gestation in women. J Lipid Res. 1996;37(2):299-308.

20. Merzouk H, Meghelli-Bouchenak M, Loukidi B, Prost J, Belleville J. Impaired serum lipids

and lipoproteins in fetal macrosomia related to maternal obesity. Biol Neonate. 2000;77(1):17-24.

21. Kontush A, Therond P, Zerrad A, et al. Preferential sphingosine-1-phosphate enrichment and sphingomyelin depletion are key features of small dense HDL3 particles: relevance to antiapoptotic and antioxidative activities. Arterioscler Thromb Vasc Biol. 2007;27(8):1843-1849.

22. Davidson WS, Silva RA, Chantepie S, Lagor WR, Chapman MJ, Kontush A. Proteomic analysis of defined HDL subpopulations reveals particle-specific protein clusters: relevance to antioxidative function. Arterioscler Thromb Vasc Biol. 2009;29(6):870-876.

23. Kontush A, Chantepie S, Chapman MJ. Small, dense HDL particles exert potent protection of atherogenic LDL against oxidative stress. Arterioscler Thromb Vasc Biol. 2003;23(10):1881-1888.

24. Martin SS, Jones SR, Toth PP. High-density lipoprotein subfractions: current views and clinical practice applications. Trends Endocrinol Metab. 2014;25(7):329-336.

25. Hart B, Morgan E, Alejandro EU. Nutrient sensor signaling pathways and cellular stress in fetal growth restriction. J Mol Endocrinol. 2019;62(2):R155-R165.

Figure legends

Fig. 1 Maternal serum HDL-c, HDL2-c, and HDL3-c concentrations and HDL2-c/HDL3-c ratios in the first trimester in the AGA and LGA groups. a Maternal serum HDL-c, HDL2-c, and HDL3-c

concentrations in the first trimester in the AGA and LGA groups. **b** The ratio of HDL2-c/HDL3-c in the AGA and LGA groups. AGA, appropriate for gestational age. LGA, large for gestational age. **P* < 0.05, ***P* < 0.01.

Fig. 2 The incidence of LGA based on different maternal HDL-c, HDL2-c, and HDL3-c concentrations and HDL2-c/HDL3-c ratios in the first trimester. a The incidence of LGA based on different maternal HDL-c concentrations in the first trimester. **b** The incidence of LGA based on different maternal HDL2-c concentrations in the first trimester. **c** The incidence of LGA based on different maternal HDL3-c concentrations in the first trimester. **d** The incidence of LGA based on different maternal ratios of HDL2-c/HDL3-c in the first trimester. LGA, large for gestational age. AGA, appropriate for gestational age. **P* value < 0.05, ***P* value < 0.01.

Fig. 3 Nomogram for the risk of LGA. To estimate the probability of LGA, the values of a pregnant woman value were marked at each axis. A straight line was drawn perpendicular to the point axis, and the points for all variables were summed. Next, the sum was noted on the total point axis, and a straight line was drawn perpendicular to the probability axis. LGA, large for gestational age. GWG, gestational weight gain. BMI, body mass index.

Fig. 4 Receiver operating characteristic curve for the prediction model. The area under the curve (AUC) was 0.663 (95% CI 0.593-0.732).

Table legends

Table 1 Maternal and neonatal characteristics

All data are expressed as the mean ± standard deviation or number (percentage). AGA, appropriate for gestational age. LGA, large for gestational age. BMI, body mass index. [a] Statistical significance of the difference between the AGA and LGA categories using the independent sample t test for continuous variables and Pearson's chi-square test for categorical variables.

Table 2 Kendall's tau_b correlations between the incidence of LGA and HDL-c, HDL2-c, and HDL3-c concentrations and the ratio of HDL2-c/HDL3-c

LGA, large for gestational age. $^*P < 0.05$, $^{**}P < 0.01$.

Table 3 The association between maternal HDL2-c concentration in the first trimester and risk of LGA

The model was adjusted for maternal age, GWG, prepregnancy BMI and parity. GWG, gestational weight gain. BMI, body mass index.

20220803105359752159393072443392

ORIGINALITY REPORT

28%

SIMILARITY INDEX

PRIMARY SOURCES

1	lipidworld.biomedcentral.com<br>Internet	158 words — 4%
2	www.incor.usp.br<br>Internet	94 words — 2%
3	www.science.gov<br>Internet	92 words — 2%
4	core.ac.uk<br>Internet	65 words — 2%
5	www.researchsquare.com<br>Internet	62 words — 1%
6	Hongliang Wang, Qinyu Dang, Haiyan Zhu, Ning Liang, Zhiyin Le, Dongxu Huang, Rong Xiao, Huanling Yu. "Associations between maternal serum HDL-c concentrations during pregnancy and neonatal birth weight: a population-based cohort study", Lipids in Health and Disease, 2020<br>Crossref	46 words — 1%
7	Ning Liang, Haiyan Zhu, Xueping Cai, Zhiyin Le, Hongliang Wang, Dian He, Rong Xiao, Huanling Yu. "The high maternal TG level at early trimester was associated with the increased risk of LGA newborn in non-obesity pregnant women", Lipids in Health and Disease, 2018	44 words — 1%

Crossref

8	www.thieme-connect.com<br>Internet	33 words — 1%
9	cardiab.biomedcentral.com<br>Internet	27 words — 1%
10	www.pubfacts.com<br>Internet	26 words — 1%
11	Harun Kilic, Enver Atalar, Incilay Lay, Nuray Yazihan et al. "High-density lipoprotein subfractions and influence of endothelial lipase in a healthy Turkish population: A study in a land of low high-density lipoprotein cholesterol", Scandinavian Journal of Clinical and Laboratory Investigation, 2014<br>Crossref	25 words — 1%
12	bmcpregnancychildbirth.biomedcentral.com<br>Internet	25 words — 1%
13	pdffox.com<br>Internet	25 words — 1%
14	www.jlr.org<br>Internet	25 words — 1%
15	academic.oup.com<br>Internet	24 words — 1%
16	Ruo-Lin Hou, Huan-Huan Zhou, Xiao-Yang Chen, Xiu-Min Wang, Jie Shao, Zheng-Yan Zhao. "Effect of maternal lipid profile, C-peptide, insulin, and HBA1c levels during late pregnancy on large-for-gestational age newborns", World Journal of Pediatrics, 2014<br>Crossref	23 words — 1%

17	assets.researchsquare.com<br>Internet	22 words — 1%
18	link.springer.com<br>Internet	20 words — < 1%
19	Giuliano Generoso. "Associação entre as subfrações de colesterol da lipoproteína de alta densidade mensuradas pelo método de Perfil Vertical Automático e síndrome metabólica, inflamação, resistência à insulina e risco de doença vascular subclínica: Estudo Longitudinal de Saúde do Adulto", Universidade de Sao Paulo, Agencia USP de Gestao da Informacao Academica (AGUIA), 2021<br>Crossref Posted Content	19 words — < 1%
20	wrap.warwick.ac.uk<br>Internet	19 words — < 1%
21	Baba, Amuda. "Strengthening the Midwifery Workforce in Fragile Contexts: a Mixed Methods Study from Ituri Province, Democratic Republic of Congo.", The University of Liverpool (United Kingdom), 2021<br>ProQuest	18 words — < 1%
22	cyberleninka.org<br>Internet	18 words — < 1%
23	worldwidescience.org<br>Internet	18 words — < 1%
24	www.ijms.info<br>Internet	15 words — < 1%
25	Kelley, G.A.. "Aerobic exercise and HDL"2-C: A meta-analysis of randomized controlled trials", Atherosclerosis, 200601	13 words — < 1%

Crossref

---

26 Xiafang Wu, Chenchen Wei, Ruifeng Chen, Linxian Yang, Weifei Huang, Liang Huang, XinXin Yan, Xuedong Deng, Zhongshan Gou. "Fetal umbilical artery thrombosis: prenatal diagnosis, treatment and follow-up", Research Square Platform LLC, 2022 — 12 words — < 1%
Crossref Posted Content

---

27 bmjpaedsopen.bmj.com — 12 words — < 1%
Internet

---

28 etheses.bham.ac.uk — 12 words — < 1%
Internet

---

29 www.coloproctol.org — 12 words — < 1%
Internet

---

30 bmcwomenshealth.biomedcentral.com — 11 words — < 1%
Internet

---

31 www.karger.com — 11 words — < 1%
Internet

---

32 Anatol Kontush, M. John Chapman. "Functionally Defective High-Density Lipoprotein: A New Therapeutic Target at the Crossroads of Dyslipidemia, Inflammation, and Atherosclerosis", Pharmacological Reviews, 2006 — 10 words — < 1%
Crossref

---

33 Joao Alveiro Alvarado Rincón, Jorgea Pradieé, Mariana Härter Remião, Tiago Veiras Collares et al. " Effect of high-density lipoprotein on oocyte maturation and bovine embryo development ", Reproduction in Domestic Animals, 2018 — 10 words — < 1%
Crossref

34 Kayla L. Dobson, Danilo F. da Silva, Sheila Dervis, Shuhiba Mohammad, Taniya S. Nagpal, Kristi B. Adamo. "Physical activity and gestational weight gain predict physiological and perceptual responses to exercise during pregnancy", Birth Defects Research, 2020 — 10 words — < 1%
Crossref

35 Koh, Yunsuk. "The effects of niacin and a single bout of exercise on blood lipid and lipoprotein profiles in postmenopausal women", Proquest, 20111004 — 10 words — < 1%
ProQuest

36 Wei Bao, Sharon Dar, Yeyi Zhu, Jing Wu, Shristi Rawal, Shanshan Li, Natalie L. Weir, Michael Y. Tsai, Cuilin Zhang. "Plasma concentrations of lipids during pregnancy and the risk of gestational diabetes mellitus: A longitudinal study", Journal of Diabetes, 2018 — 10 words — < 1%
Crossref

37 acta.uta.fi — 10 words — < 1%
Internet

38 f1000research.com — 10 words — < 1%
Internet

39 orca.cf.ac.uk — 10 words — < 1%
Internet

40 Gianni Biolo, Filippo G. Di Girolamo, Adam McDonnell, Nicola Fiotti et al. "Effects of Hypoxia and Bed Rest on Markers of Cardiometabolic Risk: Compensatory Changes in Circulating TRAIL and Glutathione Redox Capacity", Frontiers in Physiology, 2018 — 9 words — < 1%
Crossref

41 bmcendocrdisord.biomedcentral.com — 9 words — < 1%
Internet

42 germany.omicsonline.org
Internet
9 words — < 1%

43 www.jmir.org
Internet
9 words — < 1%

44 www.researchgate.net
Internet
9 words — < 1%

45 Gugliucci, Alejandro, Russell Caccavello, Kazuhiko Kotani, Naoki Sakane, and Satoshi Kimura. "Enzymatic assessment of paraoxonase 1 activity on HDL subclasses: A practical zymogram method to assess HDL function", Clinica Chimica Acta, 2013.
Crossref
8 words — < 1%

46 iosrjournals.org
Internet
8 words — < 1%

47 www.bmrat.org
Internet
8 words — < 1%

48 Cong Chen, Qiuyu Feng, Mengtong Yang, Sijia Chen, Hong Sun, Yiqi Zhang, Shihan Pu, Hong Chen, Danping Su, Yishan Guo, Guo Zeng. "Maternal HDL-c levels are associated with preterm birth and small for gestational age: A prospective study in China", Research Square Platform LLC, 2022
Crossref Posted Content
7 words — < 1%

49 Handbook of Experimental Pharmacology, 2015.
Crossref
7 words — < 1%

50 Ito, Yasuki, Noriyuki Satoh, Takayoshi Ishii, Junko Kumakura, and Tsutomu Hirano. "Development of a homogeneous assay for measurement of high-density lipoprotein-subclass cholesterol", Clinica Chimica Acta, 2014.
7 words — < 1%

Crossref

51 Vytautas Žėkas, Rėda Matuzevičienė, Dovilė Karčiauskaitė, Dalius Vitkus et al. "Changes in circulating endothelial microvesicles in men after myocardial infarction", Advances in Medical Sciences, 2020 — 6 words — < 1%
Crossref

52 www.ncbi.nlm.nih.gov — 6 words — < 1%
Internet

EXCLUDE QUOTES	OFF	EXCLUDE SOURCES	OFF
EXCLUDE BIBLIOGRAPHY	ON	EXCLUDE MATCHES	OFF
